# Effectiveness of steroids versus placebo in preventing upper airway obstruction after extubation in critically ill children: rationale and design of a multicentric, double-blind, randomized study

**DOI:** 10.1186/s13063-020-4218-2

**Published:** 2020-04-19

**Authors:** Gema Manrique, Laura Butragueño-Laiseca, Rafael González, Corsino Rey, Zuriñe Martínez de Compañon, Javier Gil, Antonio Rodríguez-Núñez, Cecilia Martínez, Silvia Manrique, Jesús López-Herce

**Affiliations:** 1 Pediatric Intensive Care Unit, Gregorio Marañón General University Hospital, Calle Doctor Castelo 47, 28009 Madrid, Spain; 2 Gregorio Marañón, Health Research Institute, Pabellón de Gobierno. Doctor Esquerdo 46, 28007 Madrid, Spain; 3 Maternal and Child Health and Development Research Network (Red SAMID), Madrid, Spain; 4 Department of Public Health and Pediatrics, Complutense University of Madrid (Spain), Plaza Ramon y Cajal s/n., 28040 Madrid, Spain; 5 Pediatric Intensive Care Unit, Central Hospital of Asturias, Avenida de Roma s/n, 33011 Oviedo, Spain; 6 Pediatric Intensive Care Unit, Vall d’Hebron University Hospital, Passeig de la Vall d’Hebron 119-129, 08035 Barcelona, Spain; 7 Pediatric Intensive Care Unit, Cruces University Hospital, Plaza de Cruces s/n, 48903 Barakaldo, Spain; 8 Pediatric Intensive Care Unit, University Hospital of Santiago de Compostela, Rua da Choupana, 15706 Santiago de Compostela, Spain; 9 Pharmacy Unit, Gregorio Marañón General University Hospital, Calle Doctor Castelo 47, 28009 Madrid, Spain

**Keywords:** Steroids, Dexamethasone, Stridor, Upper airway obstruction, Children, Extubation

## Abstract

**Background:**

Post-extubation upper airway obstruction (UAO) is a frequent complication causing stridor and respiratory distress, which occasionally require reintubation, thereby increasing morbidity and mortality rates. Contradictory results have been obtained in studies assessing the effectiveness of steroids in preventing post-extubation UAO, and the available evidence is limited. We designed a multicentric randomized, placebo-controlled study to explore the effectiveness of dexamethasone in preventing post-extubation UAO in children.

**Methods:**

A multicentric, prospective, double-blind, randomized, placebo-controlled, phase IV clinical trial has been designed. The sample will include pediatric patients who are between 1 month and 16 years of age and who have been intubated for more than 48 h. Patients who have airway disorders or who have received steroids within the previous seven days will be excluded. Patients will be randomly assigned to receive either placebo or a therapy with dexamethasone 0.25 mg/kg every 6 h to be started 6 to 12 h prior to extubation (to a total of four doses). Randomization will be performed at a 1:1 ratio. Follow-up of patients will be carried out for 48 h after extubation. The main objective of this study is to access the reduction in the incidence of moderate to severe UAO symptoms following extubation. Secondary objectives include assessing the decrease in the incidence of reintubation, evaluating the use of additional therapies for UAO, and monitoring potential side effects of dexamethasone.

**Discussion:**

The results of this study will contribute to the existing evidence on prophylaxis for post-extubation airway obstruction.

**Trial registration:**

EudraCT identifier: 2009–016596-30. Registered on May 11, 2010.

## 1. Background

Critically ill pediatric patients frequently require endotracheal intubation. Post-extubation upper airway obstruction (UAO) is a common complication that affects up to a third of patients who require endotracheal intubation [1–3].

Owing to the small diameter of their airways, laryngeal edema is more frequent and severe in children than in adults. Indeed, this type of edema in children affects the subglottal region more frequently than the glottal region [2, 4]. Signs of airway obstruction include stridor and respiratory distress, which require reintubation in 6–13% of patients [1–3].

Risk factors related to post-extubation UAO include underlying respiratory or neurological disease, prolonged intubation (more than 36–48 h), reintubation, a young age (≤24 months of age), or infant weight of less than 5 kg [1, 2].

The obstruction of the upper airway has significant effects on the evolution of the patient. This complication may increase morbidity and mortality, length of hospital stay, duration of mechanical ventilation, and health care–related costs, especially when the patient requires reintubation [1, 2, 5, 6].

Steroids are among the most widely used therapies for post-extubation UAO, as they have anti-inflammatory effects, thereby reducing the risk for laryngeal edema and airway obstruction. A range of steroids such as hydrocortisone, methylprednisolone, and dexamethasone have been used for the prevention and management of post-extubation UAO [4, 7]. Contradictory results have been obtained in studies assessing the effectiveness of steroids in preventing post-extubation UAO [5–10].

A recent meta-analysis [7] of studies in adult patients with or without UAO risk factors receiving different regimens of steroids showed that steroids reduce the incidence of upper airway complications and reintubation in high-risk patients.

Studies in pediatric patients are based on widely heterogeneous samples of patients and doses and timing of prophylactic therapy ranging from 1 h to 24 h prior to extubation [4, 7].

In studies on the use of dexamethasone in children, a variety of regimens have been used versus placebo. Some authors report a decrease in the incidence of stridor [5, 8], whereas two studies show a reduction in the incidence of reintubation [8, 9]. A study in neonates concluded that dexamethasone reduced the risk for reintubation [10]. In contrast, this benefit has not been proven in other studies in children [11] and neonates [12].

Baranwal et al. [3] compared two regimens in high-risk pediatric patients: starting dexamethasone therapy 6 h versus 24 h before extubation. The authors found that the 24-h regimen, as compared with the 6-h regimen, significantly reduced the incidence of UAO.

A Cochrane’s systematic review [4] concluded that the effectiveness of steroids in preventing UAO in children has not been sufficiently demonstrated, and further studies are needed to assess its potential benefits, especially in high-risk patients.

In light of the lack of consistent evidence, we designed a randomized study to compare the effectiveness of dexamethasone versus placebo in the prevention of post-extubation UAO in high-risk pediatric patients (>48 h intubated).

The main objective of this study is to explore whether dexamethasone is effective in preventing and reducing the severity of UAO symptoms in critically ill children versus placebo. Secondary objectives include investigating whether dexamethasone reduces the incidence of reintubation and evaluating the potential secondary effects associated with this medication.

## 2. Methods

### 2.1. Study design

This is a multicentric, prospective, double-blind, randomized, placebo-controlled, phase IV clinical trial. Five pediatric intensive care units of hospitals in Spain will participate in the study. The study protocol was developed in accordance with the Standard Protocol Items: Recommendations for Interventional Trials (SPIRIT) checklist (Additional file 1). In May 2010, the study was registered with the European Clinical Trials Register (number 2009–016596-30) under the name “Steroids in the prevention of upper airway postextubation complications in critically ill children”.

### 2.2. Study population

The sample will include children (between 1 month and 16 years of age) who are admitted to the intensive care unit and who require intubation for more than 48 h, regardless of their condition. Patients with airway malformations, suspected or confirmed croup syndrome, tracheitis, or epiglottitis and those who had previously undergone any surgery involving upper or lower airway will be excluded. Other exclusion criteria are the administration of steroid therapy within the previous seven days, patients who had a previous extubation failure during the stay, or refusal to participate in the study.

### 2.3. Recruitment

**Fig. 1:**
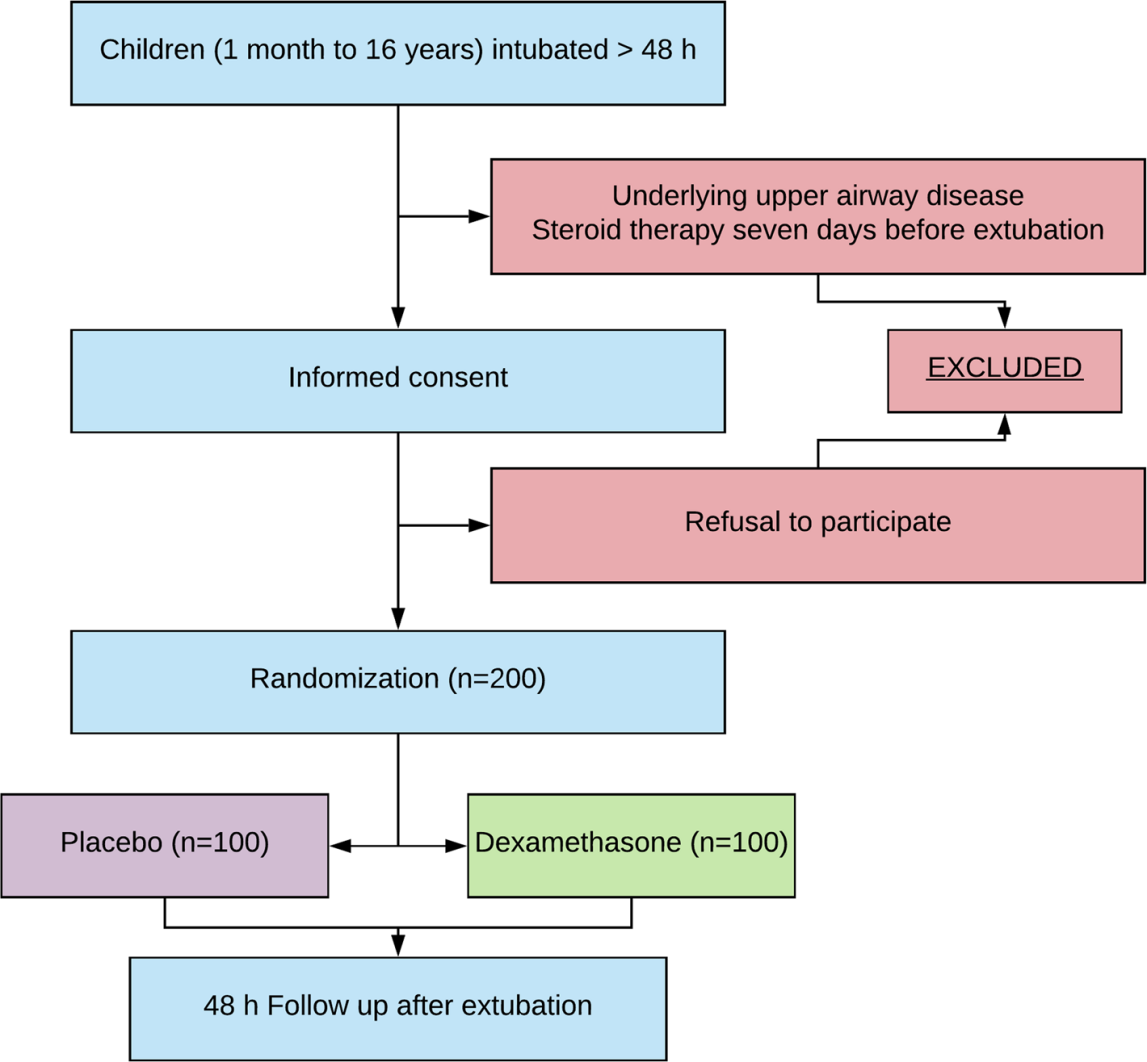
CONSORT (Consolidated Standards of Reporting Trials) flow diagram

Study candidates will be identified by a study physician, who will explain the study to parents or guardians. Written informed consent will be obtained from parents or guardians prior to inclusion in the study. A CONSORT (Consolidated Standards of Reporting Trials) flow diagram is shown in Fig. 1.

### 2.4. Randomization and masking

Patients will be assigned to one of the two therapy groups on a 1:1 ratio by simple randomization using a randomization table on EPIDAT 3.1 (Epidat: software package for epidemiological data analysis. 2006. Consellería de Sanidade, Xunta de Galicia, España; Organización Panamericana de la Salud; Universidad CES, Colombia). The coordinating center will send a table for sequential randomization of patients. This table will contain the number of the medication that has been assigned to each patient. The treatment group will not be detailed in the randomization table. This table will include a number of a reserve medication for use in case of deterioration or loss or if the drug is rendered unusable. The pharmacy unit of the coordinating center will send treatment-arm assignments labeled and blinded as established in the randomization table for each center.

The labeling and blinding of medication kits will be performed by the pharmacy unit of the coordinating center, which will keep an open record of these kits and their composition. This record will be coded and stored by the coordinating center pharmacist responsible for the labeling and submission of samples.

If severe or unexpected adverse events related to the medication appear the principal investigator will require the appointed pharmacist to unblind the codes identifying the sample. The principal investigator will be informed so that timely action can be taken. The unblinding of any code will be recorded by the pharmacist responsible for sample blinding.

### 2.5. Intervention

The treatment group will receive intravenous 0.25 mg/kg per dose (to a maximum of 8 mg) every 6 h for a total of four doses. The first dose will be administered between 6 and 12 h prior to extubation. The placebo group will be administered saline 0.9%. The study medication will have the same aspect and characteristics as those of placebo and both will be administered in the same way. Dose adjustment, discontinuance, or reinitiation of treatment will not be allowed. Inspections will be performed to ensure that the treatment is being administered adequately.

### 2.6. Data collection and assessment of efficacy

On inclusion, demographic (age, sex, and weight) and clinical data will be collected as follows:

- Diagnosis (six diagnostic groups will be established): surgery, lower airway obstruction, neurological disease, sepsis, trauma, and other diagnosis.
- Assessment of severity: the clinical status of the patient will be assessed by using Pediatric Index of Mortality 2 (PIM2), Pediatric Risk of Mortality III (PRISM 3), and Pediatric Logistic Organ Dysfunction (PELOD) scores prior to extubation.
- Size and type of endotracheal tube (cuffed or uncuffed).
- Route of intubation (oral or nasal).
- Previous need for endotracheal tube replacement.
- Previous airway endoscopies.
- Respiratory infection confirmed by endotracheal aspirate culture.
- Presence of blood in endotracheal aspirate.
- Duration of endotracheal intubation prior to extubation (in days).

The primary endpoint is the reduction of the incidence of moderate to severe UAO symptoms within 48 h after extubation. Moderate to severe UAO symptoms will be considered if stridor or a Taussig score of more than 5 is present. The secondary endpoints are the presence of reintubation and the potential secondary effects associated with dexamethasone.

A record of the following variables will be kept at 15 min and 1, 2, 6, 12, 24, and 48 h after extubation:

1. UAO Taussig score [13], a clinical scoring system for the assessment of UAO severity; evaluating stridor, retractions, inflow of air into the lungs, cyanosis, and consciousness (Table 1). Presence of inspiratory stridor.
2. Need for and frequency of additional therapies for respiratory distress: adrenaline or nebulized budesonide, intravenous steroids, heliox, or non-invasive ventilation.
3. Hemodynamic (blood pressure and heart rate) and respiratory (peripheral oxygen saturation and respiratory rate) parameters.
4. Arterial partial pressure of carbon dioxide (pCO_2_) and partial pressure of oxygen (pO_2_) and glycemia. Blood samples will not be routinely collected; they will be drawn only for clinical purposes.
5. Need for reintubation, time point, and cause.
6. Presence of digestive bleeding.
7. Occurrence of infection.

**Table 1:** SPIRIT (Standard Protocol Items: Recommendations for Interventional Trials) Schedule of Events Timeline: enrolment, assessment of safety, assessment of efficacy, and dispensing of the medicine

Tests	Screening	First visit prior to extubation	Visits 2 to 8
**Assessment of eligibility**			
Informed consent	X		
Inclusion and exclusion criteria	X		
Anamnesis	X		
**Assessment of safety**			
Physical examination	X	X	X
Vital signs	X	X	X
Assessment of adverse events		X	X
Previous and current medications	X	X	X
**Assessment of efficacy**			
Vital signs	X	X	X
Stridor	X	X	X
Taussig scale	X	X	X
Reintubation		X	X
**Other assessments**			
Other medications		X	X
**Deliveries of medication**			
Randomization and dispensing of the medication		X	X
Contact with the randomized-dispensing center	X	X	X

Visits 2 to 8, respectively: 15 min, 1 h, 2 h, 6 h, 12 h, 24 h, and 48 h after extubation

The timing of parameter recording is shown in Table 2.

**Table 2:** Modified Taussig score

	Score			
	0	1	2	3
Clinical parameters				
Color	Normal	Normal	Normal	Cyanotic
Air entry	Normal	Mildly diminished	Moderately diminished	Substantially diminished
Retractions	None	Mild	Moderate	Severe
Level of consciousness	Normal	Restlessness if disturbed	Restlessness at rest	Lethargy
Stridor	None	Mild	Moderate	Severe (or no stridor if severe obstruction)

Upper airway obstruction is considered mild if score is less than 5 points, moderate-severe with at least 5 points. Modified Taussig score [13]

All interventions that patients require for their treatment are allowed but those that could affect the upper airway (respiratory tract infections, reintubations, etc.) will be recorded and will be taken into account in the statistical analysis.

For each participating center, a co-investigator will be appointed to verify the correct adherence to the study protocol, checking in every 8-h shift to confirm that the medication is prescribed in the treatment and that it has been administered. This investigator will also be responsible for complete follow-up of every patient.

A monthly communication newsletter regarding recruitment activity will be sent by email to all researchers. In this newsletter, researchers will also be encouraged to continue recruiting.

### 2.7. Evaluation of safety

The occurrence of adverse events from inclusion to study completion will be recorded for all patients. All patients, including those who discontinue their participation in the study, will be monitored until hospital discharge. Severe adverse events during hospital stay will be reported to the study coordinator and promotor. If severe adverse events occur, follow-up will be performed until remission or until a diagnosis is made and its association with the study medication is established.

A severe adverse event is defined as any adverse event that the patient’s treating physician considers to require treatment on the basis of the characteristics and clinical status of the patient. Severe hyperglycemia is any glycemia greater than 200 mg/dL that persists for more than 6 h or physicians consider to require treatment on the basis of the clinical status of the patient (or both).

Any unused medication will be disposed of in accordance with the protocol established by the pharmacy unit in collaboration with the unit of environmental management of the coordinating center, in compliance with ISO 14.001. Upon study completion, any unused or partially used medication will be sent back to the coordinating center.

Sample size

The estimated incidence of moderate to severe UAO symptoms is approximately 33% [1–3]. We consider a clinically relevant result to be a 50% reduction in the incidence of moderate to severe UAO symptoms in the treatment group.

To calculate the minimum sample size, a two-sample two-proportion two-tiled comparison test was used, and arcsine was used for the approximation for proportions (Cohen’s method). To detect a 50% reduction of incidence (33% UAO symptoms proportion in the placebo group and 16% expected proportion in the treatment group; that is, Cohen’s h effect size = 0.4) with 80% statistical power and a 5% significance level, 110 subjects per arm will be needed. A follow-up loss rate of 10% has been estimated.

### 2.8. Statistics

Categorical variables will be expressed as frequencies and percentages. Normality of continuous data will be assessed by the Kolmogorov–Smirnov test. Continuous variables will be expressed as mean values and standard deviation or as median values and interquartile range.

To compare the primary endpoint of outcome between the group receiving dexamethasone and the group receiving placebo, Z-test statistics will be used. Per-protocol analysis will be performed. An intention-to-treat analysis will be performed to guarantee the effect of randomization.

The association between other categorical variables will be assessed by chi-squared test or Pearson’s coefficient or by Fisher’s exact test, as appropriate, based on the sample size. Continuous variables will be compared by Student’s *t* test or Mann–Whitney *U* test. In comparisons of values with respect to time, Student’s *t* test of repeated measures or Wilcoxon test will be performed, as appropriate. Additional subgroup analysis according to age and pathologies will be performed.

Bilateral statistical tests will be used at a 5% level of significance. Statistical analysis will be performed by using SPSS version 20 (SPSS Inc., Chicago, IL, USA).

### 2.9. Data processing and auditing

Data from each patient will be collected and anonymized by a study member in a data log. Each study center will send their data logs to the coordinating center. The principal investigator will enter data from data logs into a single database for all study patients. An interim analysis will be performed by an independent data monitoring committee composed of members of the Central Unit of Clinical Research Support and Clinical Trials (UCAICEC) of the Gregorio Marañón Research Institute. The study will be interrupted only if the incidence of UAO or of severe adverse events is significantly higher in the steroid treatment group as compared with the placebo group. The final analysis will be carried out when the sample size has been reached. The UCAICEC will be responsible for auditing the trial independently from investigators yearly.

## 3. Discussion

Post-extubation UAO is a common complication that affects up to a third of patients, causing stridor and respiratory distress that require reintubation in 6–13% of patients [1–3]. There is no conclusive evidence of the effectiveness of steroids in preventing post-extubation UAO in pediatric patients [4]. If steroids were proven to be effective, they would become the standard treatment for those patients. This would help reduce morbidity and mortality, the length of hospital stay, the duration of mechanical ventilation, and hospital costs [1, 2, 5, 6].

On the basis of the limited evidence currently available, we designed a multicentric, randomized, double-blind study. The primary endpoints are post-extubation UAO symptoms, as they reflect the effects of steroids adequately. Reintubation is the most severe consequence of extubation failure and is the factor with the most significant impact on prognosis [2, 4]. Yet, as the incidence of reintubation is low, the number of patients needed for detecting differences between treatment groups would be very high. In addition, the recruitment of pediatric patients of these characteristics, who are often critically ill, is challenging. Therefore, the reduction in the incidence of reintubation, together with the occurrence of adverse events, was established as a secondary objective.

Prolonged reintubation is one of the most significant risk factors for reintubation [1, 5]. Considering the evidence provided in the literature [1, 3, 4, 11], we decided to use intubation for more than 48 h as an inclusion criterion. We included other risk factors reported to be associated with UAO [1, 2], namely tube size and characteristics (cuffed or uncuffed), underlying respiratory diseases, and previous manipulation of the airway. We decided to exclude patients who have received corticosteroids within the previous seven days given that the biological half-life of dexamethasone is 36–54 h and the elimination is completed (94%) after 4 half-lives.

Steroids are used for the management of multiple diseases. Although they involve some risks, the administration of four doses of steroids for prophylaxis of the UAO after extubation is not expected to cause significant adverse events [4, 7, 11]. Therefore, the potential benefit of steroids would outbalance its associated risks.

The steroid most widely used for the prevention of post-extubation complications is dexamethasone [4, 7]. For this reason, we selected this medication for our study. However, there is variability in the timing of steroid administration prior to extubation. The intervals used range from 1 h prior to extubation to 24 h [4, 7]. In several studies, the administration of dexamethasone started 12 h prior to extubation [5, 7–9]. We decided to start the prophylactic therapy 6–12 h before extubation. This way, it is not necessary to delay extubation until the prophylactic treatment has been completed. Delayed extubation in the final stage of mechanical ventilation withdrawal is associated with a higher risk for trauma in the upper airways as a result of tube displacement when the patient moves or secondary to a more frequent need of secretion aspiration.

It is important that the study sample be composed of the patients who are more likely to benefit from steroids, as their use involves certain side effects such as hyperglycemia, hypertension, infection, and digestive bleeding [4, 7].

The most significant limitation of this study is the high number of patients needed. The number of participating centers is not very high, and completion of the study is expected to take a long time. Indeed, as it occurs in other trials with critically ill children, the percentage of inclusion is expected to be low because of difficulties in recruiting critically ill children. Another limitation of this study is the occurrence of other factors with impact on post-extubation respiratory failure (neurologic, cardiac, or respiratory diseases), which may hinder the assessment of a UAO. Moreover, it may be difficult to distinguish adverse events related to steroids (hyperglycemia and digestive bleeding) from events related to the underlying disease and critical state of patients. The results of this study will contribute to the existing evidence on prophylaxis for post-extubation UAO.

### 3.1. Trial status

Protocol version: V02, July 19, 2010. Recruitment to this study commenced in February 2013. The interim analysis was performed by the data monitoring committee in April 2017. Patient recruitment is expected to be finished during the first half of 2020. To date, more than 120 patients have been recruited. For public or scientific queries, contact Jesús López-Herce by using the contact information provided in the affiliations of this article.

## Supplementary information

**Additional file 1.**

**Additional file 2.**

## Data Availability

Recruitment has commenced and data will not be released until the end of the trial (once the main analyses have been completed and the primary outcome manuscript published). Trial data (including full protocol trial dataset) will be available on a public data repository once the trial has finished.

## Abbreviations

UAO: Upper airway obstruction
UCAICEC: Central Unit of Clinical Research Support and Clinical Trials
