# Supplementary material for: Effectiveness of steroids versus placebo in preventing upper airway obstruction after extubation in critically ill children: rationale and design of a multicentric, double-blind, randomized study
Source: Trials. 2020 Apr 19;21:341. doi: 10.1186/s13063-020-4218-2 (PMC7168970; doi:10.1186/s13063-020-4218-2)
Supplement: Supplementary file 2 — Additional file 2. [file 13063_2020_4218_MOESM2_ESM.pdf]

HOJA DE INFORMACIÓN AL PACIENTE Y CONSENTIMIENTO INFORMADO PARA LOS PADRES/TUTORES DEL NIÑO

TÍTULO DEL ESTUDIO: CORTICOIDES PARA LA PREVENCIÓN DE LAS COMPLICACIONES DE LA VIA AÉREA POSTEXTUBACIÓN EN NIÑOS CRÍTICOS

CÓDIGO DEL PROMOTOR: FIBHGM-ECNC003-2010(FIBHGM-10-01)

PROMOTOR: FUNDACIÓN PARA LA INVESTIGACIÓN BIOMËDICA DEL HOSPITAL GREGORIO MARAÑÓN

INVESTIGADOR PRINCIPAL: JESÚS LÓPEZ-HERCE CID

CENTRO: HOSPITAL GENERAL UNIVERSITARIO GREGORIO MARAÑÓN DE MADRID

INTRODUCCION

Nos dirigimos a usted para informarle sobre un estudio de investigación en el que se le invita a participar. El estudio ha sido aprobado por el Comité Ético de Investigación Clínica correspondiente y la Agencia Española del Medicamento y Productos Sanitarios, de acuerdo a la legislación vigente, el Real Decreto 223/2004, de 6 de febrero, por el que se regulan los ensayos clínicos con medicamentos. Nuestra intención es tan solo que usted reciba la información correcta y suficiente para que pueda evaluar y juzgar si quiere o no participar en este estudio. Para ello lea esta hoja informativa con atención y nosotros le aclararemos las dudas que le puedan surgir después de la explicación. Además, puede consultar con las personas que considere oportuno.

PARTICIPACIÓN VOLUNTARIA

Debe saber que su participación en este estudio es voluntaria y que puede decidir no participar o cambiar su decisión y retirar el consentimiento en cualquier momento, sin que por ello se altere la relación con su médico ni se produzca perjuicio alguno en su tratamiento.

DESCRIPCIÓN GENERAL DEL ESTUDIO

La intubación endotraqueal es una práctica habitual en las Unidades de Cuidados Intensivos Pediátricos. Sin embargo, ésta técnica no está exenta de riesgos y tras la extubación hasta un 35% de los niños presentan dificultad para respirar por inflamación de la vía aérea (laringe y tráquea). Algunas veces si la dificultad respiratoria es importante es preciso volver a intubar y esto aumenta las complicaciones y prolonga la estancia en la unidad de cuidados intensivos.

Los corticoides podrían disminuir la inflamación de la vía aérea. Sin embargo, los estudios realizados hasta ahora no han demostrado claramente si los corticoides son útiles para evitar estas complicaciones de la intubación.
Se solicita que usted autorice incluir a su hijo en un estudio de investigación clínica en el que se utiliza un fármaco (dexametasona) que está comercializado para otras enfermedades desde hace muchos años y se utiliza, para evaluar si la dexametasona evitaría la obstrucción de la vía aérea postextubación en los niños ingresados en cuidados intensivos.

OBJETIVO DEL ESTUDIO

El propósito de este estudio es valorar la eficacia de la dexametasona en la prevención de la obstrucción de la vía aérea postextubación en niños ingresados en cuidados intensivos pediátricos así como estudiar los efectos secundarios del tratamiento.

PROCEDIMIENTOS DEL ESTUDIO

Si acepta que su hijo participe en el estudio, se le incluirá al azar en uno de los dos grupos de tratamiento. La probabilidad de que a su hijo se le incluya en uno u otro grupo es del 50%. En el primer grupo de tratamiento los niños recibirán dexametasona. En segundo grupo los niños recibirán un placebo (un líquido sin ningún efecto). En todos los niños se vigilará de forma continua la aparición de dificultad respiratoria tras la extubación y los posibles efectos secundarios tomando las medidas oportunas.
Tanto las extracciones de sangre para las analíticas como la administración de la medicación intravenosa, se realizarán en general a través de un catéter previamente colocado por lo que no le producirán ningún dolor a su hijo

BENEFICIOS Y RIESGOS DERIVADOS DE SU PARTICIPACIÓN EN EL ESTUDIO

Es posible que su hijo no obtenga ningún beneficio para su salud por participar en este estudio. También es posible que en su hijo tenga menor riesgo de presentar dificultad para respirar tras la extubación y disminuya las posibilidades de tenerle que reintubar.
La participación en este estudio puede ayudar a conocer si las corticoides son eficaces para evitar los problemas de la vía aérea que pueden aparecer tras la extubación en los niños ingresados en cuidados intensivos pediátricos

El fármaco utilizado, la dexametasona, se utiliza durante muchos años en niños. Este puede producir en ocasiones efectos no deseados que generalmente son leves y

transitorios (ceden al suspender el fármaco). Los más frecuentes son el aumento de azúcar en sangre (hiperglucemia) y de la tensión arterial. También puede existir un mayor riesgo de sangrado gástrico y por ello, todos serán tratados con medicamentos que protegen el estómago. En todos los niños que se incluyan en el estudio se vigilará la aparición de estos efectos y si se producen se valorará la suspensión del fármaco.

El médico responsable del estudio le podrá informar de todas las dudas que le surjan.

SEGURO

El Promotor del estudio dispone de una póliza de seguros que se ajusta a la legislación vigente y que le proporcionará la compensación e indemnización en caso de menoscabo de su salud o de lesiones que pudieran producirse en relación con su participación en el estudio.

CONFIDENCIALIDAD

El tratamiento, la comunicación y la cesión de los datos de carácter personal de todos los sujetos participantes se ajustará a lo dispuesto en la Ley Orgánica 15/1999, de 13 de diciembre de protección de datos de carácter personal. De acuerdo a lo que establece la legislación mencionada, usted puede ejercer los derechos de acceso, modificación, oposición y cancelación de datos, para lo cual deberá dirigirse a su médico del estudio.

Los datos recogidos para el estudio estarán identificados mediante un código y solo su médico del estudio/colaboradores podrán relacionar dichos datos con usted y con su historia clínica. Por lo tanto, su identidad no será revelada a persona alguna salvo excepciones (si existe alguna situación especial por la que se necesitara conocer la identidad del sujeto para cumplir con algún requisito del estudio se debe explicar en este apartado), en caso de urgencia médica o requerimiento legal.

Sólo se transmitirán a terceros y a otros países los datos recogidos para el estudio que en ningún caso contendrán información que le pueda identificar directamente, como nombre y apellidos, iniciales, dirección, nº de la seguridad social, etc. En el caso de que se produzca esta cesión, será para los mismos fines del estudio descrito y garantizando la confidencialidad como mínimo con el nivel de protección de la legislación vigente en nuestro país.

El acceso a su información personal quedará restringido al médico del estudio/colaboradores, autoridades sanitarias (Agencia Española del Medicamento y Productos Sanitarios), al Comité Ético de Investigación Clínica y personal autorizado por el promotor, cuando lo precisen para comprobar los datos y procedimientos del

estudio, pero siempre manteniendo la confidencialidad de los mismos de acuerdo a la legislación vigente.

COMPENSACIÓN ECONÓMICA

El promotor del estudio es el responsable de gestionar la financiación del mismo. Para la realización del estudio el promotor del mismo ha firmado un contrato con el centro donde se va a realizar y con el médico del estudio.

Usted no tendrá que pagar por los medicamentos del estudio.

OTRA INFORMACIÓN RELEVANTE

Cualquier nueva información referente a los fármacos utilizados en el estudio y que pueda afectar a su disposición para participar en el estudio, que se descubra durante su participación, le será comunicada por su médico lo antes posible.

Si usted decide retirar el consentimiento para participar en este estudio, ningún dato nuevo será añadido a la base de datos y, puede exigir la destrucción de todas las muestras identificables previamente retenidas para evitar la realización de nuevos análisis.

También debe saber que puede ser excluido del estudio si el promotor los investigadores del estudio lo consideran oportuno, ya sea por motivos de seguridad, por cualquier acontecimiento adverso que se produzca por la medicación en estudio o porque consideren que no está cumpliendo con los procedimientos establecidos. En cualquiera de los casos, usted recibirá una explicación adecuada del motivo que ha ocasionado su retirada del estudio Al firmar la hoja de consentimiento adjunta, se compromete a cumplir con los procedimientos del estudio que se le han expuesto.

Cuando acabe su participación recibirá el mejor tratamiento disponible y que su médico considere el más adecuado para su enfermedad, pero es posible que no se le pueda seguir administrando la medicación del estudio. Por lo tanto, ni el investigador ni el promotor adquieren compromiso alguno de mantener dicho tratamiento fuera de este estudio.

ESTUDIOS CLÍNICOS EN MENORES DE EDAD

Le informamos que al tratarse de la participación de su hijo que tiene más de 12 años de edad, se le va a entregar a él mismo una hoja de información y consentimiento informado adaptados a su capacidad de entendimiento y deberá firmarlos. Además, de acuerdo a la legislación vigente, el promotor del estudio ha puesto en conocimiento del Ministerio Fiscal que se van a incluir menores de edad en el mismo.

DOCUMENTO DE CONSENTIMIENTO INFORMADO DEL SUJETO PARTICIPANTE

Código del Estudio:

Yo (nombre y apellidos)

....

He leído la hoja de información que se me ha entregado.

He podido hacer preguntas sobre el estudio.

He recibido suficiente información sobre el estudio.

He hablado con:

....

(nombre del investigador)

Comprendo que mi participación es voluntaria.

Comprendo que puedo retirarme del estudio:

1º Cuando quiera

2º Sin tener que dar explicaciones.

3º Sin que esto repercuta en mis cuidados médicos.

- Presto libremente mi conformidad para participar en el estudio y doy mi consentimiento para el acceso y utilización de mis datos en las condiciones detalladas en la hoja de información.

Firma del paciente: Firma del investigador:

Nombre: Nombre:

Fecha: Fecha:

DOCUMENTO DE CONSENTIMIENTO INFORMADO DEL REPRESENTANTE LEGAL

Código del Estudio: FIBHGM-ECNC003-2010(FIBHGM-10-01)

Yo (nombre y apellidos) …………………………………………..en calidad de………………….(relación con el participante) de……….………………………………..(nombre y apellidos del participante)

He leído la hoja de información que se me ha entregado.

He podido hacer preguntas sobre el estudio.

He recibido suficiente información sobre el estudio.

He hablado con: ....(nombre del investigador)

Comprendo que la participación del paciente es voluntaria.

Comprendo que puede retirarse del estudio:

1º Cuando quiera

2º Sin tener que dar explicaciones.

3º Sin que esto repercuta en sus cuidados médicos.

- En mi presencia se ha dado a……………………………………….....(nombre del participante) toda la información pertinente adaptada a su nivel de entendimiento y está de acuerdo en participar. Presto mi conformidad para que …………………………………………………(nombre del participante) participe en este estudio y doy mi consentimiento para el acceso y utilización de los datos en las condiciones detalladas en la hoja de información.

Firma del representante:	Firma del investigador:
Nombre:	Nombre:
Fecha:	Fecha:

DOCUMENTO DE CONSENTIMIENTO INFORMADO ORAL ANTE TESTIGOS

Código del Estudio: FIBHGM-ECNC003-2010(FIBHGM-10-01)

Yo……………………………………………(nombre y apellidos del testigo) declaro bajo mi

responsabilidad que …………………………….. …….(nombre y apellidos del participante)

Ha leído (ó se le ha leído, en el caso en que el paciente no pueda leer), la hoja de información

que se le ha entregado.

Ha podido hacer preguntas sobre el estudio.

Ha recibido suficiente información sobre el estudio.

Ha hablado con: ....(nombre del investigador)

Comprende que su participación es voluntaria.

Comprende que puede retirarse del estudio:

1º Cuando quiera

2º Sin tener que dar explicaciones.

3º Sin que esto repercuta en sus cuidados médicos.

- Ha expresado libremente su conformidad para participar en el estudio en este estudio y da para el acceso y utilización de los datos en las condiciones detalladas en la hoja de información.

Firma del testigo: Firma del investigador:

Nombre: Nombre:

Fecha: Fecha:
